# Temperature Changes between Neighboring Days and Mortality in Summer: A Distributed Lag Non-Linear Time Series Analysis

**DOI:** 10.1371/journal.pone.0066403

**Published:** 2013-06-24

**Authors:** Hualiang Lin, Yonghui Zhang, Yanjun Xu, Xiaojun Xu, Tao Liu, Yuan Luo, Jianpeng Xiao, Wei Wu, Wenjun Ma

**Affiliations:** 1 Guangdong Provincial Institute of Public Health, Guangzhou, China; 2 Center for Disease Control and Prevention of Guangdong Province, Guangzhou, China; The Ohio State University, United States of America

## Abstract

**Background:**

Many studies have shown that high temperatures or heat waves were associated with mortality and morbidity. However, few studies have examined whether temperature changes between neighboring days have any significant impact on human health.

**Method:**

A distributed lag non-linear model was employed to investigate the effect of temperature changes on mortality in summer during 2006–2010 in two subtropical Chinese cities. The temperature change was defined as the difference of the current day’s and the previous day’s mean temperature.

**Results:**

We found non-linear effects of temperature changes between neighboring days in summer on mortality in both cities. Temperature increase was associated with increased mortality from non-accidental diseases and cardiovascular diseases, while temperature decrease had a protective effect on non-accidental mortality and cardiovascular mortality in both cities. Significant association between temperature changes and respiratory mortality was only found in Guangzhou.

**Conclusion:**

This study suggests that temperature changes between neighboring days might be an alternative temperature indicator for studying temperature-mortality relationship.

## Introduction

It is well-known that ambient temperature is associated with fluctuations in mortality and morbidity over time [1], [2]. There has been an increasing interest in studying this relationship as a response to the climate change caused by increased greenhouse gases emissions [3]–[6].To investigate this association using time-series data, researchers have used various indicators of temperature, including mean, minimum and maximum ambient temperatures, diurnal temperature range (DTR), extremely hot or cold temperatures [1], [5], [7]–[9]. Typically, J-, V-, or U-shaped associations between temperature and mortality have been observed in previous studies in the last two decades [10]–[12].

Along with the continuing climate change, unstable weather patterns (e.g., sharp drop/increase in temperature) are projected to occur more frequently in the coming decades [13], [14], and have become important issues in public health agenda in recent years [15]. Sudden temperature changes may represent a risk factor to human health, mainly to individuals with an existing chronic condition. Therefore, temperature change between the neighboring days might be another temperature indicator for analyzing temperature impacts on human health [10]. However, to date there have been few studies to examine the health effects of temperature changes between neighboring days [16], [17]. It is well known that the effects of temperature can persist for a few days or even longer. Exposure to extreme temperature of the current day may impact the mortality of several days later [18]. Recently, a new model, namely distributed lag non-linear model has been proposed to simultaneously investigate the delayed effects and the non-linear exposure–response relationship [19]. The effects of temperature on mortality may vary with population characteristics, geographical conditions (i.e., climatic condition, vegetation), health care access and population adaptation (i.e., usage of air conditioning) [20]. To date, limited evidence is available from China about the health effects of temperature changes between adjacent days. Better understanding of this association will provide useful information for developing public health intervention programs and prevention measures that will better target on those most vulnerable to sharp temperature changes in China.

In the present study, we examined the short-term effect of temperature changes between adjacent days on mortalities from non-accidental diseases, cardiovascular and respiratory diseases in two subtropical cities of Guangdong Province, China. It is hypothesized that sharp temperature changes have significant adverse effects on human health. As the temperature-mortality relationship usually varies with seasons, we only examined the relationship between temperature changes and mortality in summer seasons (from May to September).

## Materials and Methods

### Study Setting

**Figure 1:**
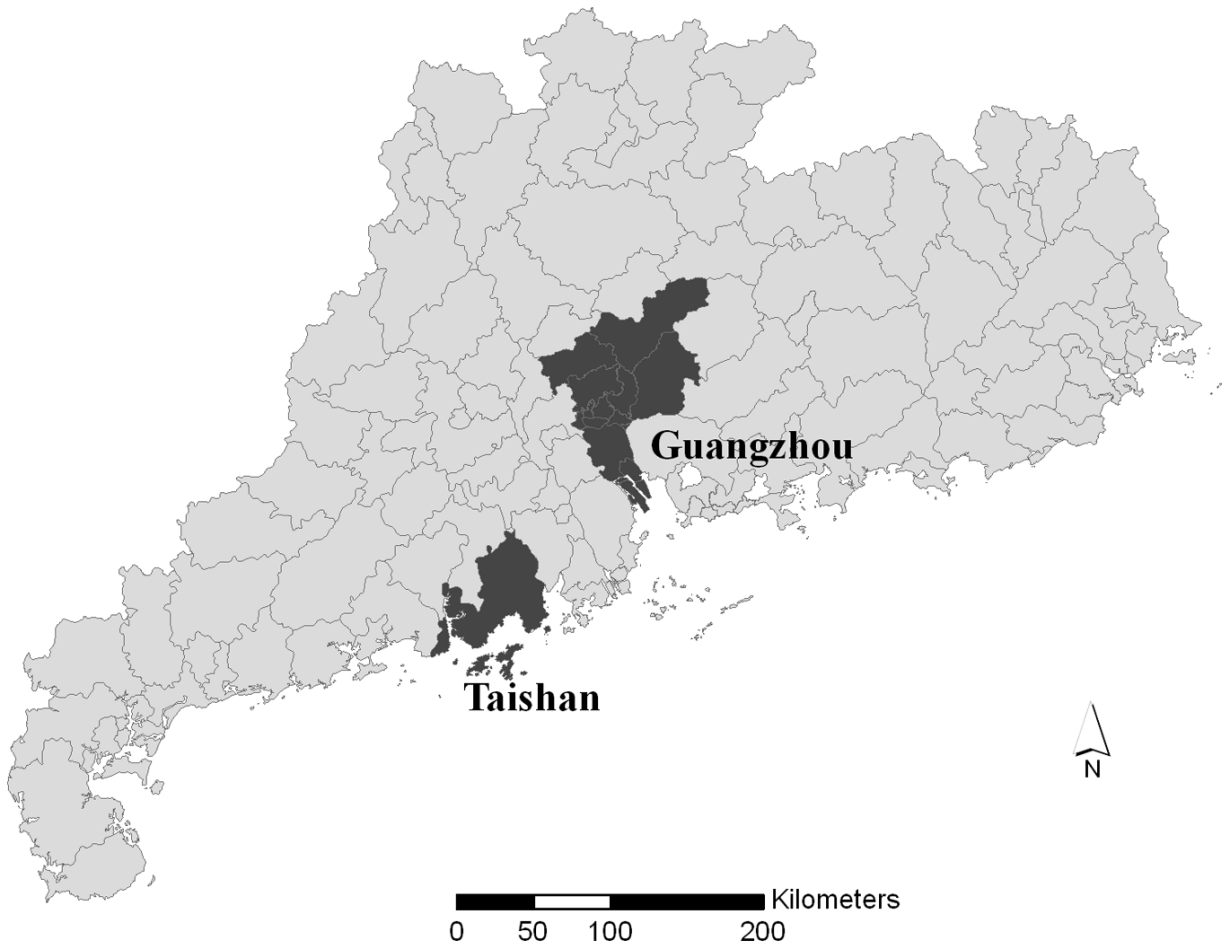
Location of the study area in Guangdong Province, China.

Guangdong Province, located in southeastern China, has a typical subtropical climate with an average annual temperature of 22°C. Residents from two cities in Guangdong Province, Guangzhou and Taishan as illustrated in Figure 1, were selected as the study area. Guangzhou is the capital city of Guangdong Province and the third largest city in China, whereas Taishan is relatively small with limited industrial activities. Both cities have a typical monsoon-influenced climate with wet and hot summers and dry and cool to mild winters [21]. Taishan has a population of about 1 million. The residents of two districts in Guangzhou City (Yue Xiu and Li Wan) were selected as the study population. These two districts combined have an area of 92.9 km^2^ and are home to 1.9 million residents. These two districts were chosen for two reasons. First, there are daily air pollution monitoring data available to this study, which were collected from three air monitoring stations in the two districts. Second, because most of those living in these two districts are permanent residents; and the mortality data are also of high quality.

### Data Collection

Daily non-accidental mortality data covering the period from January 1st 2006 to December 31st 2010 were collected from the Center for Disease Control and Prevention of Guangdong Province (GDCDC) for the two cities. GDCDC is the government agency in charge of health data collection in Guangdong Province. In Guangdong Province, a death must be reported to GDCDC; the death could occur in a hospital or at home. In both situations, the hospital or community/village doctors fill in a standard Death Certificate Card. The information is then reported to GDCDC through their internal network reporting system. The causes of death were coded according to the 10th revision of the International Classification of Diseases (ICD-10). The mortality data were classified into deaths due to all non-accidental causes (ICD-10: A00-R99), cardiovascular diseases (ICD-10: I00-I99), and respiratory diseases (ICD-10: J00-J99).

Daily meteorological data for both cities were obtained from Guangdong Meteorological Bureau for the same period. The variables included daily mean temperature (°C.), relative humidity (%) and atmospheric pressure (hpa). Temperature change was defined as the difference between the current day’s and previous day’s mean temperature. Daily 24-hour average ambient air pollution data were collected from Environmental Monitoring Center. Air pollution data included particular matter with an aerodynamic diameter less than or equal to 10 µm (PM_10_, in ug/m^3^), sulfur dioxide (SO_2_, in ug/m^3^), nitrogen dioxide (NO_2_, in ug/m^3^) and ozone (O_3_, in ug/m^3^). Daily air pollution data were measured continuously at environmental monitoring sites located in the centers of Guangzhou and Taishan, respectively.

### Statistical Analysis

As the count of daily mortality typically followed a Poisson distribution, a distributed lag non-linear model (DLNM) was used to simultaneously investigate the non-linear and delayed effects of temperature change on daily mortality [19], [22], [23]. In brief, we used quasi-likelihood Poisson regression in a generalized linear model to model the natural logarithm of daily counts of deaths as functions of predictor variables. This model used a “cross-basis” function that examine a two-dimensional relationship along the dimensions of temperature change and lag days. In “cross-basis” function, we used the spline function for temperature changes and the polynomial function for the lag structure. We initially conducted a “primary” model and then we did sensitivity analysis to investigate the robustness of the effect estimates. The “primary” model has a natural cubic spline with 3 df in the lag space and a cubic b-spline with 5 df in the temperature change space. We used lags up to 28 days according to the previous study to capture the overall effects [16]. Potential confounding factors were controlled for in the model, which included an indicator for day of week (DOW), an indicator for public holiday (PH), a natural spline for day of the year (DOY, with df 4/year, as we only analyzed the data for summer) in order to control the seasonal effect within each year, a smooth function of mean temperature on the current day (Temp_0_, 3 df), a smooth function of the moving average for the previous 3 days’ temperature (Temp_1–3_, 3 df), a smooth function of relative humidity (3 df) and smooth function for various air pollutants (3 df), including PM_10_, SO_2_, NO_2_ and O_3_. The model used for the analysis is:
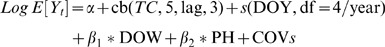
where E(Yt) denotes the expected daily mortality count on day t, cb means the “cross-basis” function, s(·) indicates a smooth function based on natural splines for nonlinear variables, β is regression coefficient, and COVs are the potential confounding factors.

We reported the relative risk (RR, with 95% confidence intervals (CIs)) of large temperature increase/drop (1%, 5%, 95% and 99% percentiles of temperature changes) on mortality along specific lag days with 0°C temperature change as the reference value. The sex-specific effect of temperature changes on total non-accidental mortality was also examined for the two cities. We also examined the cumulative effect of temperature changes on mortality along different lag days.

Because the risk estimates usually vary with the model specifications in time-series analysis [22], [24], [25], we performed additional sensitivity analyses: use of alternative degrees of freedom (3, 5 df/year) for temporal adjustment and use of alternative degrees of freedom (4, 5, and 6) for other weather variables and air pollutants. We also used alternative indicator for temperature change by the difference of the current day’s and the previous day’s maximum temperatures. In order to check whether the observed effects can be attributed to high temperature, we compared the effects estimates from models with or without daily mean temperature being included.

All statistical tests were two-sided and values of P<0.05 were considered statistically significant. The dlnm package [19] in R software Version 2.15.1 (R Development Core Team, 2012) was utilized to fit all the models.

## Results

Table 1 illustrated the distribution of daily weather conditions, air pollutants, and mortality in summer months in the two cities. There were, on average, 30.19 daily non-external deaths in Guangzhou, and 17.64 in Taishan, respectively. The temperature change ranged from −5.5°C to 4.2°C in Guangzhou, and from -5.9°C to 3.7°C in Taishan. The mean temperature was higher in Guangzhou (28.19°C) than in Taishan (27.52°C), and Taishan had higher relative humidity than Guangzhou (80.85% vs. 74.33%). Mean concentration of PM_10_, SO_2_, NO_2_ and O_3_ were 53.65 ug/m^3^, 46.82 ug/m^3^, 47.55 ug/m^3^ and 39.71 ug/m^3^ in Guangzhou and 31.92 ug/m^3^, 14.91 ug/m^3^, 14.51 ug/m3 and 21.95 ug/m^3^ in Taishan, respectively.

**Table 1:** Summary statistics of daily weather conditions, air pollutants and mortality in Guangzhou and Taishan, China.

City	Variable	Mean(SD)	Min	P25	P50	P75	Max
Guangzhou							
	Total (all non-accidental)	30.19(6.18)	15.00	26.00	30.00	34.00	54.00
	Cardiovascular	9.57(3.39)	2.00	7.00	9.00	12.00	23.00
	Respiratory	5.49(2.51)	1.00	4.00	5.00	7.00	18.00
	Temperature change (°C)	0.03(1.45)	−5.50	−0.70	0.20	1.00	4.20
	Temperature (°C)	28.19(2.33)	20.40	26.60	28.20	30.20	33.50
	Humidity (%)	74.33(9.78)	44.00	67.00	74.00	82.00	99.00
	PM_10_ (ug/m^3^)	53.65(26.10)	8.33	34.21	49.13	66.96	177.33
	SO_2_ (ug/m^3^)	46.82(24.56)	2.44	29.72	44.77	61.66	150.53
	NO_2_ (ug/m^3^)	47.55(23.69)	12.96	30.99	40.83	59.44	158.83
	O_3_ (ug/m^3^)	39.71(26.34)	0.92	18.53	35.65	54.25	154.50
Taishan							
	Total (all non-accidental)	17.64(4.76)	6.00	14.00	17.00	21.00	33.00
	Cardiovascular	10.00(3.41)	1.00	8.00	10.00	12.00	23.00
	Respiratory	2.12(1.55)	0.00	1.00	2.00	3.00	9.00
	Temperature change (°C)	0.02(1.30)	−5.90	−0.60	0.20	0.80	3.70
	Temperature (°C)	27.52(2.00)	21.00	26.20	27.50	29.10	31.60
	Humidity (%)	80.85(7.91)	46.00	76.00	80.00	87.00	99.00
	PM_10_ (ug/m^3^)	31.92(35.29)	0.00	0.00	27.00	50.00	160.00
	SO_2_ (ug/m^3^)	14.91(21.29)	0.00	0.00	6.00	23.00	165.00
	NO_2_ (ug/m^3^)	14.51(16.46)	0.00	0.00	12.00	23.00	91.00
	O_3_ (ug/m^3^)	21.95(28.26)	0.00	0.00	14.00	32.00	178.00

**Figure 2:**
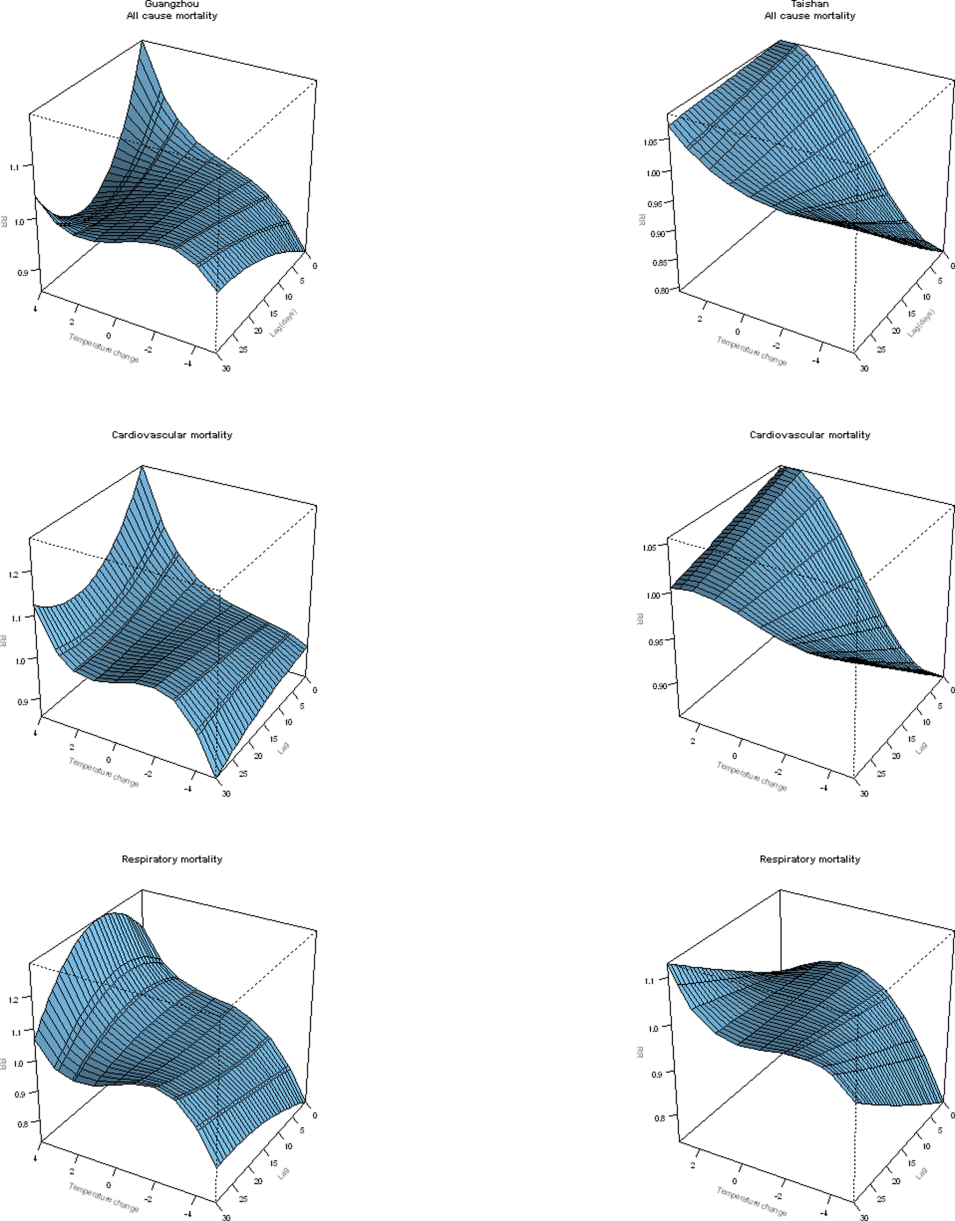
Three-D plot of RR along temperature change and lags for mortalities from non-accidental diseases, cardiovascular and respiratory diseases, with reference at 0°C temperature change.

An overall picture of the effect of temperature change on cause-specific mortality in both cities was depicted in Figure 2, showing a three-dimensional plot of the relative risk (RR) along temperature change and lags with 0°C temperature change as the reference. Overall, the estimated effects of temperature changes on mortality were non-linear. A visual inspection of the figure suggested that there was an immediate harmful effect of large temperature increase on non-accidental mortality, cardiovascular mortality and respiratory mortality (except that of Taishan), and a protective effect of temperature decrease, and the figure also suggested that there was not obvious mortality displacement for temperature increase along the lag days in both cities. For non-accidental and cardiovascular mortalities in both cities and respiratory mortality in Guangzhou, the relative risk for large temperature increase was largest on the current day and subsequently declined in the following days, and it seemed that temperature increase had a delayed effect on respiratory mortality in Taishan. Large temperature drop had biggest protective effects on the current day for all types of mortality in both cities, except cardiovascular mortality in Guangzhou.

The relative risk of daily morality by temperature change at specific lag periods (0, 5, 10, 15, and 25 days) and by lag at specific temperature changes (−4.1, −2.9, 2.1, and 2.8°C.), which corresponded to approximately the 1^st^, 5^th^, 95^th^, and 99^th^ percentiles of the temperature change distribution in Guangzhou, respectively, were illustrated in Table 2. We found that extremely high temperature decrease (−4.1°C.) was associated with decreased mortalities from non-accidental diseases, cardiovascular and respiratory diseases along the lag days, with largest effects on the current day (lag0) for non-accidental mortality (RR: 0.90, 95% CI: 0.83–0.98) and on lag 5 day for cardiovascular mortality (RR: 0.96, 95% CI: 0.74–1.00), and lag 5 day for respiratory mortality (RR: 0.85, 95% CI: 0.73–0.99), respectively. While large temperature increase was associated with elevated mortality from non-accidental diseases, cardiovascular and respiratory diseases in Guangzhou. For example, the RR for a 2.8°C. increase was 1.09 (95% CI: 1.02–1.17) for non-accidental mortality, 1.18 (95% CI: 1.03–1.37) for cardiovascular mortality on the current day, and1.11 (95% CI: 1.02–1.22) for respiratory mortality at lag 5 day, respectively.

**Table 2:** Relative risk (RR) and 95% confidence intervals (CI) for different causes of mortality for extremely temperature changes (1%, 5%, 95% and 99% percentiles) at different lag days in Guangzhou.

	RR (95% CI)				
	−4.1°C	−2.9°C	2.1°C	2.8°C	
*Death cause*					
Non-accidental					
Lag0	**0.90(0.83–0.98)**	0.95(0.90–1.01)	**1.05(1.01**–**1.10)**	**1.09(1.02**–**1.17)**	
Lag5	**0.93(0.86**–**0.99)**	0.97(0.92–1.01)	1.02(0.98–1.06)	1.04(0.98–1.10)	
Lag10	0.95(0.88–1.02)	0.98(0.93–1.03)	1.00(0.96–1.04)	1.00(0.94––1.06)	
Lag15	0.96(0.90–1.04)	0.99(0.94–1.04)	0.98(0.94–1.02)	0.98(0.92–1.04)	
Lag25	0.99(0.94–1.05)	1.01(0.98–1.05)	0.98(0.95–1.01)	0.97(0.93–1.02)	
Cardiovascular					
Lag0	0.95(0.69–1.04)	0.97(0.77–1.01)	**1.13(1.03**–**1.23)**	**1.18(1.03**–**1.37)**	
Lag5	**0.96(0.74**–**1.00)**	**0.98(0.81**–**1.00)**	1.05(0.97–1.13)	1.09(0.95–1.20)	
Lag10	0.96(0.77–1.01)	0.98(0.84–1.02)	1.03(0.92–1.08)	1.05(0.88–1.12)	
Lag15	0.96(0.80–1.03)	0.99(0.87–1.04)	1.01(0.90–1.05)	1.03(0.84–1.07)	
Lag25	0.95(0.85–1.05)	1.00(0.93–1.07)	1.00(0.92–1.05)	1.01(0.89–1.09)	
Respiratory					
Lag0	0.83(0.64–1.06)	0.92(0.78–1.09)	1.04(0.93–1.15)	1.07(0.91–1.26)	
Lag5	**0.85(0.73**–**0.99)**	0.92(0.83–1.03)	**1.07(1.01**–**1.13)**	**1.11(1.02**–**1.22)**	
Lag10	**0.87(0.77**–**0.99)**	0.93(0.87–1.01)	**1.08(1.03**–**1.14)**	**1.14(1.05**–**1.23)**	
Lag15	0.89(0.79–1.01)	0.95(0.88–1.02)	**1.08(1.02**–**1.14)**	**1.13(1.04**–**1.23)**	
Lag25	0.93(0.84–1.02)	0.99(0.93–1.05)	1.02(0.97–1.07)	1.06(0.98–1.14)	

The bold means statistically significant (p<0.05).

The relative risk of daily morality by temperature changes at specific lag periods (0, 5, 10, 15, and 25 days) and by lag at specific temperature changes (–3.6, -2.3, 2.0, and 2.6°C.), which corresponded to approximately the 1^st^, 5^th^, 95^th^, and 99^th^ percentiles of the temperature change distribution in Taishan, respectively, was depicted in Table 3. A similar pattern with that in Guangzhou was observed in Taishan. Temperature drop had an acute protective effect on both non-accidental and cardiovascular mortalities, for instance, the RR for a 3.6°C. temperature decrease on the current day was 0.87 (95% CI: 0.80–0.94) for non-accidental mortality and 0.87 (95% CI: 0.78–0.96) for cardiovascular mortality, respectively. And temperature increase was associated with increased non-accidental mortality and cardiovascular mortality with the RR 1.05 (95% CI: 1.01–1.10) for a 2.0°C. temperature increase for non-accidental mortality on the current day, and 1.05 (95% CI: 1.00–1.09) for a 2.0°C. temperature increase on lag 5 day, respectively. Different from the situation in Guangzhou, no significant association was found for respiratory mortality in Taishan.

**Table 3:** Relative risk (RR) and 95% confidence intervals (CI) for different causes of mortality for extremely temperature changes (1%, 5%, 95% and 99% percentiles) at different lag days in Taishan.

	RR (95% CI)			
	−3.6°C	−2.3°C	2.0°C	2.6°C
*Death cause*				
Non-accidental				
Lag0	**0.87(0.80–0.94)**	**0.92(0.87–0.96)**	**1.05(1.01–1.10)**	1.05(0.99–1.12)
Lag5	**0.89(0.83–0.95)**	**0.93(0.89–0.97)**	**1.04(1.01–1.08)**	**1.05(1.00–1.10)**
Lag10	**0.91(0.86–0.96)**	**0.94(0.90–0.97)**	1.04(0.92–1.07)	**1.04(1.00–1.08)**
Lag15	**0.93(0.88–0.97)**	**0.95(0.92–0.98)**	**1.03(1.01–1.06)**	**1.04(1.00–1.07)**
Lag25	0.96(0.92–1.01)	**0.97(0.94–1.00)**	**1.02(1.00–1.05)**	1.03(0.99–1.07)
Cardiovascular				
Lag0	**0.87(0.78–0.96)**	**0.9(0.85–0.96)**	1.05(0.99–1.11)	1.06(0.98–1.14)
Lag5	**0.89(0.81–0.97)**	**0.92(0.87–0.97)**	**1.05(1.00–1.09)**	1.05(0.98–1.11)
Lag10	**0.9(0.84–0.97)**	**0.93(0.89–0.97)**	**1.04(1.00–1.08)**	1.04(0.99–1.09)
Lag15	**0.92(0.87–0.98)**	**0.94(0.90–0.98)**	1.03(0.99–1.06)	1.03(0.99–1.08)
Lag25	0.96(0.91–1.02)	0.97(0.93–1.01)	1.02(0.98–1.05)	1.01(0.97–1.06)
Respiratory				
Lag0	0.87(0.69–1.09)	0.95(0.82–1.10)	0.97(0.86–1.09)	0.95(0.80–1.12)
Lag5	0.89(0.73–1.08)	0.96(0.85–1.09)	0.98(0.89–1.08)	0.97(0.85–1.12)
Lag10	0.91(0.77–1.07)	0.97(0.88–1.08)	0.99(0.92–1.08)	1(0.89–1.11)
Lag15	0.94(0.81–1.07)	0.98(0.90–1.07)	1.01(0.94–1.08)	1.02(0.93–1.12)
Lag25	0.98(0.86–1.12)	1(0.92–1.09)	1.04(0.97–1.11)	1.07(0.96–1.18)

The bold means statistically significant (p<0.05).

Table 4 showed the sex-specific relative risk of total non-accidental mortality for extremely temperature changes for specific lag days in both cities. For both sexes in Guangzhou, temperature drop was associated with acute mortality decrease, for example, the RR for a 4.1°C. temperature decrease on the current day was 0.88(95% CI: 0.79–0.97) for males on the current day and 0.90 (95% CI: 0.81–1.00) for females at lag day 5, respectively. Temperature increase was associated with elevated total non-accidental mortality for males, while no association was found for females. Similar patterns were observed in Taishan only that significant association was found for temperature increase among females, the RR for 2.0°C. temperature increase was 1.04(95% CI: 1.00–1.09) at lag day 10.

**Table 4:** Sex-specific relative risk (RR) and 95% confidence intervals (CI) for total non-accidental mortality for extremely temperature changes (1%, 5%, 95% and 99% percentiles) at different lag days in Guangzhou and Taishan.

	RR (95% CI)				
	Lag0	Lag5	Lag10	Lag15	Lag25
Guangzhou					
**Male**					
−4.1°C	**0.88(0.79–0.97)**	**0.91(0.83–0.98)**	0.93(0.86–1.02)	0.96(0.88–1.05)	1.01(0.95–1.08)
−2.9°C	**0.92(0.85–0.98)**	0.95(0.9–1.01)	0.98(0.92–1.03)	1.00(0.94–1.06)	1.02(0.98–1.06)
2.1°C	**1.08(1.02–1.14)**	1.02(0.97–1.07)	0.98(0.94–1.03)	0.96(0.92–1.01)	0.97(0.94–1.00)
2.8°C	**1.12(1.02–1.23)**	1.03(0.96–1.12)	0.98(0.9–1.06)	0.95(0.88–1.02)	0.95(0.90–1.01)
**Female**					
−4.1°C	0.92(0.8–1.04)	**0.9(0.81–1.00)**	**0.89(0.8–0.99)**	**0.89(0.8–1.00)**	0.93(0.86–1.01)
−2.9°C	0.97(0.89–1.05)	0.95(0.88–1.02)	0.94(0.87–1.01)	0.94(0.87–1.01)	0.97(0.92–1.03)
2.1°C	1.03(0.96–1.10)	1.02(0.96–1.09)	1.02(0.96–1.08)	1.01(0.95–1.08)	1.00(0.96–1.04)
2.8°C	1.06(0.94–1.18)	1.04(0.94–1.14)	1.02(0.93–1.13)	1.01(0.92–1.12)	1.00(0.94–1.07)
Taishan					
**Male**					
−3.6°C	**0.90(0.81–1.01)**	**0.92(0.84–0.99)**	0.93(0.86–1.00)	0.94(0.88–1.00)	0.96(0.90–1.02)
−2.3°C	0.94(0.88–1.00)	0.94(0.89–1.00)	**0.95(0.91–1.00)**	0.96(0.92–1.00)	0.97(0.93–1.01)
2.0°C	1.04(0.98–1.10)	1.04(0.99–1.09)	1.03(0.99–1.07)	1.03(1.00–1.06)	1.02(1.00–1.06)
2.6°C	1.05(0.97–1.14)	1.04(0.98–1.11)	**1.05(1.00–1.10)**	1.04(0.99–1.08)	1.03(0.98–1.08)
**Female**					
−3.6°C	**0.83(0.73–0.94)**	**0.86(0.77–0.95)**	**0.88(0.81–0.97)**	**0.91(0.84–0.98)**	0.97(0.90–1.04)
−2.3°C	0.89(0.82–0.96)	0.9(0.85–0.97)	0.92(0.87–0.98)	0.94(0.89–0.99)	0.97(0.93–1.02)
2.0°C	1.05(0.99–1.13)	1.05(0.99–1.11)	**1.04(1.00–1.09)**	**1.04(1.00–1.08)**	**1.03(1.00–1.07)**
2.6°C	1.06(0.96–1.16)	1.05(0.97–1.14)	1.05(0.98–1.11)	**1.05(1.00–1.10)**	1.03(0.97–1.09)

The bold means statistically significant (p<0.05).

**Figure 3:**
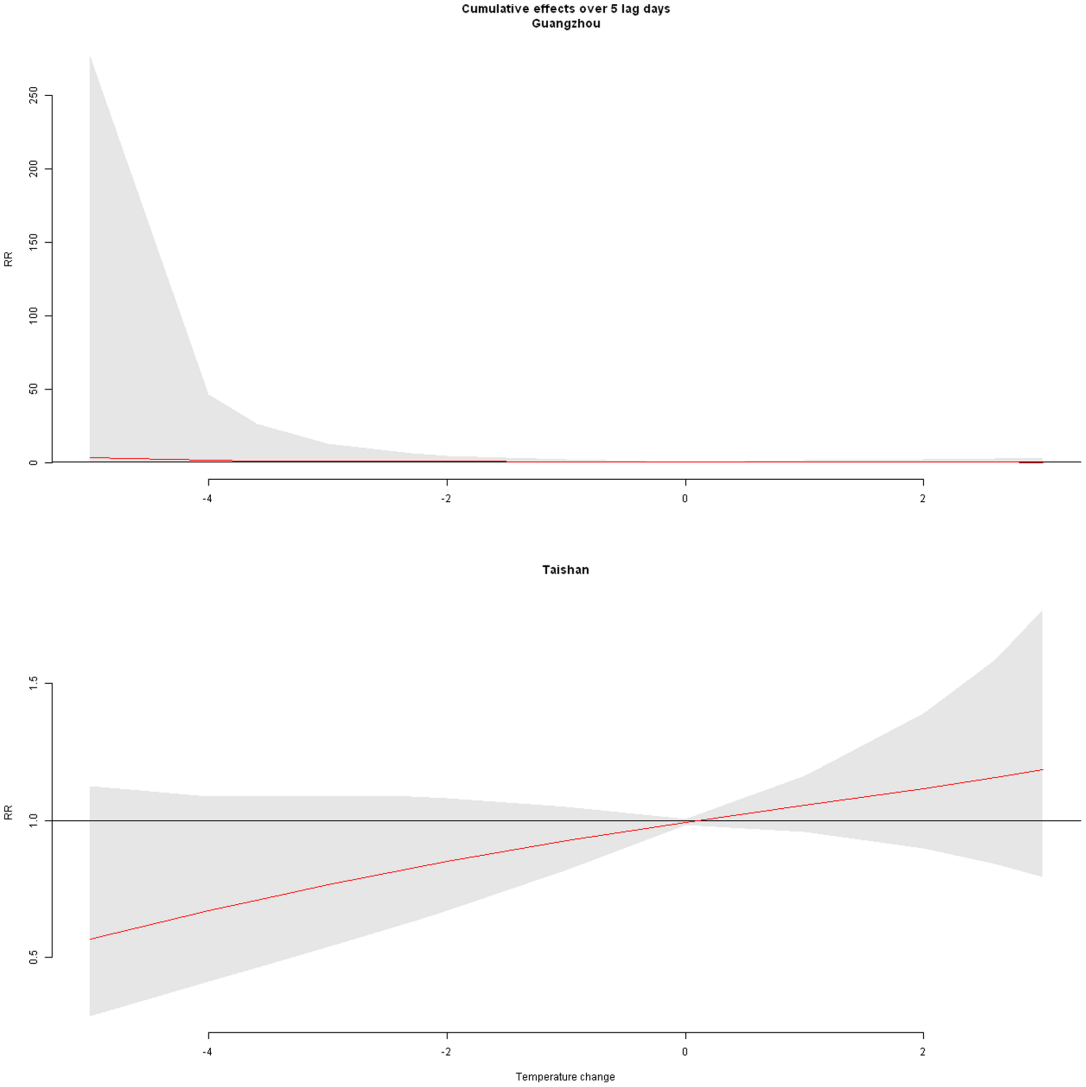
The cumulative effect of temperature changes over past 5 days on all cause mortality.

To illustrate the overall effects of temperature change along lag days on all cause mortality in the two cities, we showed the cumulative distributed non-linear lag function over 5 lag days (Figure 3). This function can be seen as the total effect of the temperature changes accumulated up to past 5 days on current day’s mortality, assuming that temperature change was the same during the past 5 days. The overall estimated RRs were 0.63 (95% CI: 0.46–0.88) and 1.31 (95% CI: 1.04–1.66) for a 4.1°C. temperature decrease and 2.8°C. increase, respectively, compared to 0°C. temperature change in Guangzhou; and the cumulative RRs in Taishan were 0.43 (95% CI: 0.33–0.56) and 1.46 (95% CI: 1.15–1.84) for a 3.6°C. temperature drop and 2.6°C. temperature increase, respectively, with 0°C. temperature change as reference.

We changed df (3, 5) for day of year to control for temporal trend, which gave similar results. We changed df (4–7) for temperature, relative humidity and various air pollutants, the estimated effects of temperature change were not substantially changed.

We used an alternative indicator for temperature change by using the difference of the current day’s and previous day’s maximum temperatures, which gave a similar results, only that it seemed that the temperature increase had a more delayed effect in Taishan (as shown in Figure S1). In addition, we compared the results from models with or without daily mean temperature being included, the effect estimates were somewhat similar (as shown in Figure S2), which meant that the observed effect of temperature changes could not be explained by high or low temperatures.

## Discussion

The present study investigated the effect of temperature change between neighboring days on mortality in two southern cities of China. Large temperature decrease between neighboring days was found to be followed by significantly decreased both all cause mortality and cardiovascular mortality, and temperature increase was associated with increased risk of mortality from non-accidental and cardiovascular diseases in both cities. The finding of this study also suggested that large temperature increase was associated with increased respiratory mortality and temperature decrease was related with decreased respiratory mortality in Guangzhou. These findings implied that physiological reactions to temperature fluctuations might depend on degree of the changes and that the direction of temperature change was also important.

Our results were not confounded by temperature. Adjusting for current day’s mean temperature and moving average of previous 3 days’ temperature did not change the effect estimates, suggesting that temperature changes between adjacent days might present itself a predictor of human health.

The harmful health effects of temperature increase found in this study were generally in accordance with previous studies that showed that temperature increase (or higher temperature) conferred excess risks of daily deaths from all cause mortality and cardiovascular mortality [2], [15], [16]. This study has also found that both temperature increase and temperature drops had delayed effects on mortality, which was slightly different from previous studies, which found that high temperature had acute health effects and low temperature had more prolonged effects [26], [27].

The harmful effect of large sudden temperature increase on mortality in summer was biologically plausible. The human body regulated the heat exchange between the body and ambient temperature by physiological control [26]. When exposed to large sudden temperature increase in summer, the automatic thermoregulation system might not adapt to sudden temperature increase, particularly for people with some chronic conditions [28]. The possible underlying mechanism may be related to dehydration, salt depletion and increased surface blood circulation [29]. Schneider et al. observed that sudden changes in temperature were also associated with risk factors of human health, such as increases in blood cholesterol levels, blood pressure, plasma fibrinogen concentrations, peripheral vasoconstriction, heart rate, platelet viscosity, and reducing the immune system’s resistance [30], [31]. All the above factors were directly associated with cardiovascular function [32]. Another study showed that an increase in ambient temperature was associated with significant changes in heart rate and electrocardiography parameters in patients in a heart rehabilitation clinic in Germany [30].

With regard to the protective effect of temperature decrease, our results were consistent with a previous study in the Czech Republic [17]. In the study area, when the temperature in summer dropped by about 3°C., the temperature was about 25°C., which was an optimum ambient temperature for local population [33], and this temperature was similar to the indoor temperature level with air conditioning, so people did not need to have too much body regulation at this temperature. Nevertheless, a recent study in Brisbane, Australia showed that a large temperature drop was associated with elevated mortality in summer [16]. And Ebi, et al. analyzed the association between temperature changes (defined as at least 3°C. decrease in maximum temperature or 3°C. increase in minimum temperature) and cardiovascular and stroke morbidity in three Californian regions, and found a significant increase in morbidity for both temperature decreases and increases, with a stronger association for the oldest age groups [34]. This discrepancy might be due to differences in weather conditions, population characteristics (e.g. socio-economic status, adaptation to temperature change, racial composition and adaptive capacity), and living conditions including usage of air conditioning, as well as access to health care in different areas [35]–[37].

The similar effects among males and females in the effects of temperature change on total non-accidental mortality might be due to similar exposure and adaptation in the two cities. Whereas the temperature change was only found to be associated with respiratory mortality in Guangzhou in this study. The study in Brisbane, Australia suggested that people with cardiovascular diseases were more vulnerable to temperature changes than those with respiratory conditions [16]. Respiratory diseases are mainly caused by the immune system’s resistance to respiratory infections [11].

Global climate change has profound impacts on human health, which has been reported to be affected by fluctuations in temperature. Special public health strategies to mitigate temperature-related adverse health effects (especially extremely high temperature in summer) have been systematically planned in many countries during the past years [15]. Elucidation of the effects of temperature variability on the mortality and morbidity is important for improvement of public health. Our study suggested that temperature changes between neighboring days should be considered as another temperature indicator in studying temperature-mortality relationship and future policy-planning in this respect. This study provided valuable evidence for policy makers to better prepare local responses to mitigate the impact of short-term temperature changes on population health. Although the underlying mechanisms for the observed relationship remained unresolved, useful information for detailed public health strategies may be generated when our findings are replicated in areas with similar geographical condition and climate profile.

Our study had two major strengths. Firstly, this study investigated the effects of temperature changes on mortality in two cities in southern China using an advanced statistical approach (dlnm) [19]. The distributed lag non-linear approach can flexibly investigate the possible association of temperature changes with daily mortality and related lag pattern. Although this model was relatively complicated and had many parameter specifications, our sensitivity analyses suggested that the results of the study were robust and insensitive to the model specifications. Secondly, in the analyses we controlled for a set of potential confounding factors including daily mean temperature, relative humidity and various air pollutants, which had been related with mortality variation, which meant that our results were relatively robust. Our study was one of the few studies to examine the health effect of temperature change between adjacent days [16], [17], [34], and suggested that large temperature increase between the neighboring days might be a risk factor of mortality.

On the other hand, a few limitations should be considered when interpreting findings from our study. Firstly, this was an ecological study in design; we used environmental monitoring data to represent the exposure level to weather conditions, which might not accurately reflect the real individual exposure. Secondly, we only used data from two cities in Guangdong Province, so the findings could be difficult to be generalized to population in other areas. Thirdly, we did not control for other time-varying factors that might affect the association between temperature change and mortality, such as PM_2.5_, living condition, air conditioning, behavior activity, dietary pattern, socioeconomic status, this might be one limitation in this study. It should be also pointed out that misclassification of cause of death was possible due to diagnostic and coding errors, but there was evidence that the accuracy of diagnoses and causes of death certificates was high in the study area in recent years [38]. Furthermore, the current study only focused on the effects of temperature changes in summer months. The finding from this analysis could not be extrapolated to other months of the year.

In conclusion, our study suggests that temperature increase between neighboring days in summer is one risk factor of mortality, and temperature decrease is protective for human health. This study suggests that temperature changes between neighboring days might be an alternative temperature indicator for studying temperature-mortality relationship.

### Ethics Statement

Data was collected as part of government mandated health surveillance and analyzed anonymously so ethical approval was not needed.

## Supporting Information

Figure S1 Three-D plot of RR along temperature change and lags for mortalities from non-accidental diseases, cardiovascular and respiratory diseases, with reference at 0°C temperature change. Temperature change was defined as the difference of the current day’s and previous day’s maximum temperatures.
(TIF)

Figure S2 Three-D plot of RR along temperature change and lags for mortalities from non-accidental diseases, cardiovascular and respiratory diseases, with reference at 0°C temperature change. Results from models without mean temperature being controlled for.
(TIF)

## References

[1] Gasparrini A, Armstrong B, Kovats S, Wilkinson P (2012) The effect of high temperatures on cause-specific mortality in England and Wales. Occupational and Environmental Medicine 69: 56–61. 21389012 10.1136/oem.2010.059782

[2] Goldberg MS, Gasparrini A, Armstrong B, Valois MF (2011) The short-term influence of temperature on daily mortality in the temperate climate of Montreal, Canada. Environmental Research 111: 853–860. 21684539 10.1016/j.envres.2011.05.022

[3] Yu W, Hu W, Mengersen K, Guo Y, Pan X, et al (2011) Time course of temperature effects on cardiovascular mortality in Brisbane, Australia. Heart 97: 1089–1093. 21487126 10.1136/hrt.2010.217166

[4] Xie H, Yao Z, Zhang Y, Xu Y, Xu X, et al (2013) Short-Term Effects of the 2008 Cold Spell on Mortality in Three Subtropical Cities in Guangdong Province, China. Environ Health Perspect 121: 210–216. 23128031 10.1289/ehp.1104541 PMC3569675

[5] Lin H, Yang L, Liu Q, Wang T, Hossain SR, et al (2012) Time series analysis of Japanese encephalitis and weather in Linyi City, China. Int J Public Health 57: 289–296. 21308477 10.1007/s00038-011-0236-x

[6] Lin HL, Lu L, Tian LW, Zhou SS, Wu HX, et al (2009) Spatial and temporal distribution of falciparum malaria in China. Malar J 8: 130. 19523209 10.1186/1475-2875-8-130 PMC2700130

[7] Nielsen J, Mazick A, Glismann S, Molbak K (2011) Excess mortality related to seasonal influenza and extreme temperatures in Denmark, 1994–2010. BMC Infectious Diseases 11: 350. 22176601 10.1186/1471-2334-11-350 PMC3264536

[8] Lim YH, Hong YC, Kim H (2012) Effects of diurnal temperature range on cardiovascular and respiratory hospital admissions in Korea. Science of The Total Environment 417–418: 55–60. 10.1016/j.scitotenv.2011.12.048 22281041

[9] Tam WWS, Wong TW, Chair SY, Wong AHS (2009) Diurnal Temperature Range and Daily Cardiovascular Mortalities Among the Elderly in Hong Kong. Archives of Environmental & Occupational Health 64: 202–206. 19864223 10.1080/19338240903241192

[10] Basu R, Samet JM (2002) Relation between elevated ambient temperature and mortality: a review of the epidemiologic evidence. Epidemiologic Reviews 24: 190–202. 12762092 10.1093/epirev/mxf007

[11] Curriero FC, Heiner KS, Samet JM, Zeger SL, Strug L, et al (2002) Temperature and Mortality in 11 Cities of the Eastern United States. American Journal of Epidemiology 155: 80–87. 11772788 10.1093/aje/155.1.80

[12] Kan H, London SJ, Chen G, Zhang Y, Song G, et al (2008) Season, sex, age, and education as modifiers of the effects of outdoor air pollution on daily mortality in Shanghai, China: the Public Health and Air Pollution in Asia (PAPA) study. Environmental Health Perspectives 116: 1183. 18795161 10.1289/ehp.10851 PMC2535620

[13] Parry ML, Canziani OF, Palutikof JP, van der Linden PJ, Hanson CE (2007) Climate change 2007: impacts, adaptation and vulnerability: Intergovernmental Panel on Climate Change.

[14] Epstein PR (2005) Climate change and human health. New England Journal of Medicine 353: 1433–1436. 16207843 10.1056/NEJMp058079

[15] Ha J, Kim H (2012) Changes in the association between summer temperature and mortality in Seoul, South Korea. International Journal of Biometeorology: 1–10. 10.1007/s00484-012-0580-4 22872184

[16] Guo Y, Barnett AG, Yu W, Pan X, Ye X, et al (2011) A large change in temperature between neighbouring days increases the risk of mortality. PloS One 6: e16511. 21311772 10.1371/journal.pone.0016511 PMC3032790

[17] Plavcova E, Kysely J (2010) Relationships between sudden weather changes in summer and mortality in the Czech Republic, 1986–2005. International Journal of Biometeorology 54: 539–551. 20169367 10.1007/s00484-010-0303-7

[18] Ha J, Shin YS, Kim H (2011) Distributed lag effects in the relationship between temperature and mortality in three major cities in South Korea. Science of The Total Environment 409: 3274–3280. 21683987 10.1016/j.scitotenv.2011.05.034

[19] Gasparrini A, Armstrong B, Kenward M (2010) Distributed lag non-linear models. Statistics in Medicine 29: 2224–2234. 20812303 10.1002/sim.3940 PMC2998707

[20] Medina-Ramon M, Zanobetti A, Cavanagh DP, Schwartz J (2006) Extreme temperatures and mortality: assessing effect modification by personal characteristics and specific cause of death in a multi-city case-only analysis. Environmental Health Perspectives 114: 1331. 16966084 10.1289/ehp.9074 PMC1570054

[21] Lu L, Lin HL, Tian LW, Yang WZ, Sun JM, et al (2009) Time series analysis of dengue fever and weather in Guangzhou, China. BMC Public Health 9: 395. 19860867 10.1186/1471-2458-9-395 PMC2771015

[22] Lin H, An Q, Luo C, Pun VC, Chan CS, et al.. (2012) Gaseous air pollution and acute myocardial infarction mortality in Hong Kong: A time-stratified case-crossover study. Atmospheric Environment DOI: 10.1016/j.atmosenv.2012.08.043

[23] Liu T, Li TT, Zhang YH, Xu YJ, Lao XQ, et al.. (2012) The short-term effect of ambient ozone on mortality is modified by temperature in Guangzhou, China. Atmospheric Environment http://dx.doi.org/10.1016/j.atmosenv.2012.07.011.

[24] Gasparrini A, Armstrong B (2010) Time series analysis on the health effects of temperature: Advancements and limitations. Environmental Research 110: 633–638. 20576259 10.1016/j.envres.2010.06.005

[25] Peng RD, Dominici F, Louis TA (2006) Model choice in time series studies of air pollution and mortality. Journal of the Royal Statistical Society: Series A (Statistics in Society) 169: 179–203.

[26] Guo Y, Punnasiri K, Tong S (2012) Effects of temperature on mortality in Chiang Mai city, Thailand: a time series study. Environmental Health 11: 36. 22613086 10.1186/1476-069X-11-36 PMC3391976

[27] Lin YK, Ho TJ, Wang YC (2011) Mortality risk associated with temperature and prolonged temperature extremes in elderly populations in Taiwan. Environmental Research. 10.1016/j.envres.2011.06.008 21767832

[28] Zanobetti A, O’Neill MS, Gronlund CJ, Schwartz JD (2012) Summer temperature variability and long-term survival among elderly people with chronic disease. Proceedings of the National Academy of Sciences 109: 6608–6613. 10.1073/pnas.1113070109 PMC3340087 22493259

[29] Bouchama A, Knochel JP (2002) Heat stroke. New England Journal of Medicine 346: 1978–1988. 12075060 10.1056/NEJMra011089

[30] Schneider A, Schuh A, Maetzel FK, Ruckerl R, Breitner S, et al (2008) Weather-induced ischemia and arrhythmia in patients undergoing cardiac rehabilitation: another difference between men and women. International Journal of Biometeorology 52: 535–547. 18228048 10.1007/s00484-008-0144-9

[31] McGeehin MA, Mirabelli M (2001) The potential impacts of climate variability and change on temperature-related morbidity and mortality in the United States. Environmental Health Perspectives 109: 185–189. 11359685 10.1289/ehp.109-1240665 PMC1240665

[32] Bhaskaran K, Hajat S, Haines A, Herrett E, Wilkinson P, et al (2009) Effects of ambient temperature on the incidence of myocardial infarction. Heart 95: 1760–1769. 19635724 10.1136/hrt.2009.175000

[33] Yan QH, Zhang YH, Ma WJ, Xu YJ, Xu XJ, et al (2011) Association between temperature and daily mortality in Guangzhou,2006–2009: a time-series study. Chin J Epidemiol 32: 9–12. 21518532

[34] Ebi K, Exuzides K, Lau E, Kelsh M, Barnston A (2004) Weather changes associated with hospitalizations for cardiovascular diseases and stroke in California, 1983–1998. International Journal of Biometeorology 49: 48–58. 15138867 10.1007/s00484-004-0207-5

[35] Stafoggia M, Forastiere F, Agostini D, Biggeri A, Bisanti L, et al (2006) Vulnerability to heat-related mortality: a multicity, population-based, case-crossover analysis. Epidemiology 17: 315–323. 16570026 10.1097/01.ede.0000208477.36665.34

[36] McCarthy JJ (2001) Climate change 2001: impacts, adaptation, and vulnerability: contribution of Working Group II to the third assessment report of the Intergovernmental Panel on Climate Change: Cambridge University Press.

[37] Ostro B, Rauch S, Green R, Malig B, Basu R (2010) The effects of temperature and use of air conditioning on hospitalizations. American Journal of Epidemiology 172: 1053–1061. 20829270 10.1093/aje/kwq231

[38] Yu ITS, Zhang YH, San Tam WW, Yan QH, Xu YJ, et al (2012) Effect of ambient air pollution on daily mortality rates in Guangzhou, China. Atmospheric Environment 46: 528–535.

