# Supplementary figures and images for: Temperature Changes between Neighboring Days and Mortality in Summer: A Distributed Lag Non-Linear Time Series Analysis
Source: PLoS One. 2013 Jun 24;8(6):e66403. doi: 10.1371/journal.pone.0066403 (PMC3691212; doi:10.1371/journal.pone.0066403)

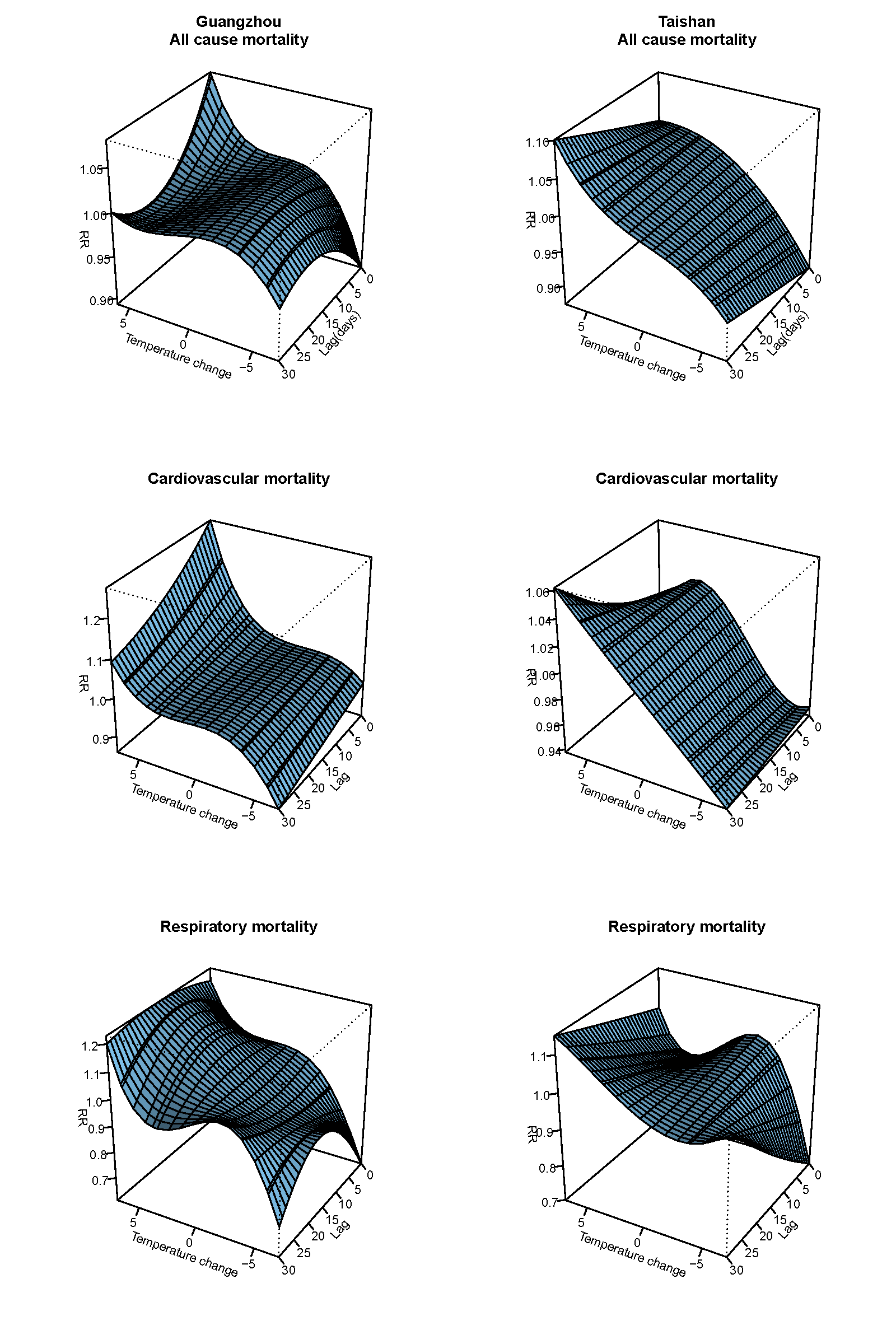

Supplement: Figure S1 — Three-D plot of RR along temperature change and lags for mortalities from non-accidental diseases, cardiovascular and respiratory diseases, with reference at 0°C temperature change. Temperature change was defined as the difference of the current day’s and previous day’s maximum temperatures. (TIF) [file pone.0066403.s001.tif]

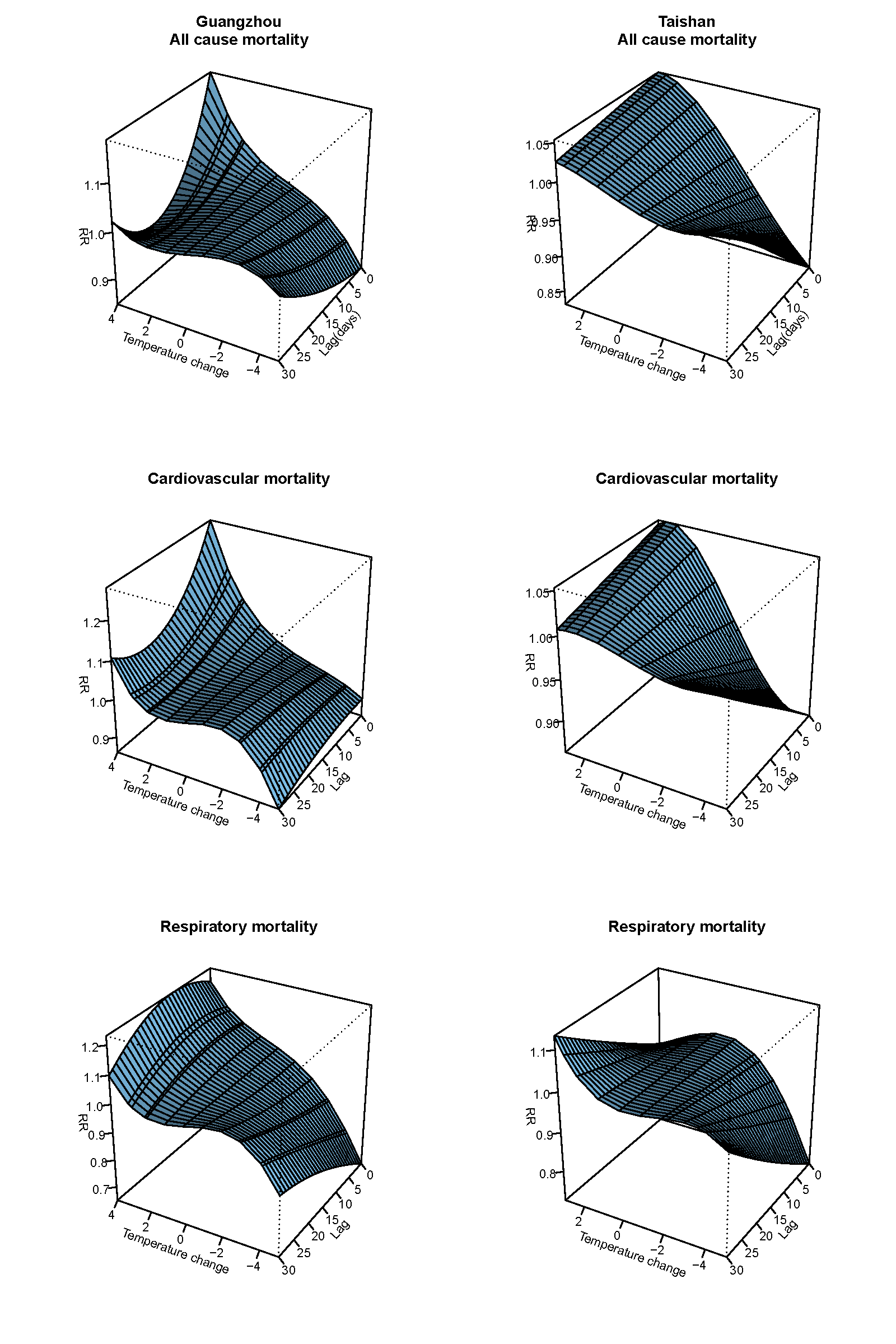

Supplement: Figure S2 — Three-D plot of RR along temperature change and lags for mortalities from non-accidental diseases, cardiovascular and respiratory diseases, with reference at 0°C temperature change. Results from models without mean temperature being controlled for. (TIF) [file pone.0066403.s002.tif]
